# Contemporary pathways to adolescent pregnancy in Indonesia: A qualitative investigation with adolescent girls in West Java and Central Sulawesi

**DOI:** 10.1371/journal.pgph.0001700

**Published:** 2023-10-27

**Authors:** Sherria Ayuandini, Marie Habito, Steven Ellis, Elissa Kennedy, Maki Akiyama, Gerda Binder, Sandeep Nanwani, Margaretha Sitanggang, Neira Budiono, Ali Aulia Ramly, Karen Humphries-Waa, Peter S. Azzopardi, Julie Hennegan

**Affiliations:** 1 PT Empatika Consultindo Mandiri, Jakarta, Indonesia; 2 Burnet Institute, Global Adolescent Health Group, Melbourne, Victoria, Australia; 3 Murdoch Children’s Research Institute, Centre for Adolescent Health, Parkville, Victoria, Australia; 4 School of Public Health and Preventive Medicine, Monash University, Melbourne, Victoria, Australia; 5 UNFPA Asia Pacific Regional Office, Bangkok, Thailand; 6 UNICEF East Asia & Pacific Regional Office, Bangkok, Thailand; 7 UNFPA Indonesia Country Office, Jakarta, Indonesia; 8 UNICEF Indonesia Country Office, Jakarta, Indonesia; 9 Telethon Kids Institute, Adolescent Health and Wellbeing, Adelaide, South Australia, Australia; 10 Melbourne School of Population and Global Health, University of Melbourne, Carlton, Victoria, Australia; University of California Berkeley School of Public Health, UNITED STATES

## Abstract

In the last decade, reduction in adolescent fertility rates in Indonesia has slowed despite national programmes and policies focused on addressing child marriage. Indonesia currently has the highest number of births to adolescent girls aged 15–19 years in Southeast Asia. There is a need to develop a more nuanced understanding of the drivers of adolescent pregnancy in Indonesia to inform programmes and policies tailored to young people’s needs and priorities. This study explored adolescent girls’ pathways to pregnancy across two provinces (Central Sulawesi and West Java) in Indonesia. We conducted participatory timeline interviews with 79 girls aged 15–21 years from urban, peri-urban, and rural communities and inquired about their relationships and life experiences leading up to pregnancy. We conducted follow-up interviews with 19 selected participants to validate and clarify preliminary findings. We identified six pathways to adolescent pregnancy which were broadly differentiated by the timing of pregnancy relative to marriage. Three pregnancy pathways within marriage were further differentiated by the main motivation for marriage–financial reasons, protecting the girl and family’s reputation, or to progress a romantic relationship. Three pregnancy pathways outside marriage were distinguished by the nature of the sexual relationship preceding pregnancy–consensual sex, unwanted or pressured sex, and forced sex. Drivers of adolescent pregnancy include the acceptability of child marriage and stigma surrounding premarital pregnancy, family and social expectations of pregnancy following marriage, harmful gender-based norms and violence, and lack of sexual and reproductive health information and access to services. Adolescents follow varied pathways to pregnancy in Indonesia. The idealisation and acceptance of child marriage is both a catalyst and outcome of adolescent pregnancy, which is occurring amid stigma surrounding premarital sex and pregnancy, harmful gender-based norms and violence, and barriers to contraceptive access and use. Our findings emphasise that there are many drivers of adolescent pregnancy and different pathways will require intervention approaches that address child marriage alongside other key contributors.

## Introduction

Pregnancy among adolescents–young people aged 110–19 years old [1]–is a public health and human rights priority. Adolescent mothers and their babies experience poorer maternal and infant health outcomes [2–5], such as higher risk of preeclampsia [6,7] and low birth weight [8–10] compared to adult mothers and their babies. Further, adolescent pregnancy can limit girls’ education and employment opportunities in later life [11,12]. Adolescent pregnancies are more likely to be unintended, as adolescents often have poor access to sexuality education and sexual and reproductive health (SRH) services, resulting in higher unmet need for contraception compared to adults [13,14]. Despite global efforts to reduce adolescent births, progress has stagnated over the past 20 years in many low- and middle-income countries (LMICs) [15]. In Southeast Asia, the adolescent fertility rate (AFR) remains at 43 births per 1,000 girls aged 15–19 years (compared to 26 in South Asia and 7 in East Asia) and many countries have seen little reduction (or increases) in these rates [16].

In Indonesia, child marriage and adolescent pregnancy are closely linked. About one in nine women aged 20–24 years in Indonesia are married by age 18, and of these women, two-thirds became pregnant by age 18 [17]. Data indicate that most women experience first sex around the time of first marriage [18,19] and Indonesian adolescents aged 15–24 years predominantly disapprove of premarital sex for girls and boys [18,19]. However, studies have shown that despite conservative ideals, premarital sex and pregnancy is common and likely underreported [20,21].

The AFR in Indonesia has been decreasing since the early 2000s (54.6 births per 1,000 girls aged 15–19 years in 2000, down to 32 in 2023), but the rate of reduction has slowed over the last decade [22]. Indonesia currently has the highest number of births to adolescent girls aged 15–19 years in Southeast Asia [22]. Recent government efforts to reduce adolescent fertility have focused on preventing child marriage, premarital sex, and drug use [23,24]. The Indonesian government passed legislation raising the minimum age of marriage for girls from 16 to 19 years, however, marriage can still occur at any age if granted an exemption (dispensation) from a religious court [17]. While changing the minimum marriage age is an important step toward addressing adolescent pregnancy within marriage, it does little to curb pregnancies before/outside marriage. Laws governing the provision of SRH services (e.g., contraceptives) remain geared toward married couples and do not explicitly address the SRH needs of unmarried people (including female and male adolescents), and abortion remains illegal with few exceptions [25–28]. Data show that majority of never-married adolescents aged 15–24 years have dating experience, many of whom have engaged in physical intimacy, with a small proportion also reporting premarital sexual experience [18]. Furthermore, recent analysis found that 26% of adolescent pregnancies were likely to have been conceived premaritally [20]. Stigma surrounding premarital sex and pregnancy may further mask premarital conceptions.

Drivers of adolescent pregnancy must be understood within their sociocultural context [29]. Heteronormative gender roles and conservative attitudes strongly disapproving of sex outside of marriage are deeply embedded within Indonesia’s state ideology and sociocultural norms [30,31]. Topics related to sex, relationships, and contraception are considered taboo, particularly among the unmarried, and young people often lack information [32–34]. The value placed on female virginity prior to marriage, and the idealization of marriage and motherhood all exert influence over the sexual behaviour of Indonesian young people [31,35]. Premarital sex and pregnancy are both highly stigmatized and a source of shame for the girl and her family [31,36]. There is a need to improve understanding of the drivers of adolescent pregnancy in their complex social context to inform programmes and policies tailored to young people’s needs and priorities.

### The present study

This study sought to understand adolescent girls’ pathways to pregnancy across two diverse provinces in Indonesia. Using participatory qualitative methods, we aimed to explore girls’ pathways to pregnancy, develop pathway typologies, and identify key drivers of pregnancy that could be targeted by policy and programming.

## Methods

### Study setting

Indonesia is made up of 38 provinces and as of the 2020 Census, has a total population of 274 million people [37]. This study was undertaken in the provinces of Central Sulawesi and West Java. Central Sulawesi is home to roughly three million people of various ethnic groups, while West Java is the most populous province in Indonesia with a total population of 48 million [37]. These provinces were selected to capture settings of high adolescent fertility and premarital conception (Central Sulawesi), and median adolescent fertility and premarital conception (West Java) based on internal data analysis by UNFPA. Within each province, we recruited girls from a mix of urban, peri-urban, and rural communities in at least two districts.

### Recruitment

We sought to recruit 60–80 adolescent girls (aged 16 to 20 years) who experienced pregnancy at age 18 or younger. Participants were sampled purposively by rurality and based on pre- or post-marital conception with the help of local facilitators and organisations providing support to pregnant adolescents. Our sampling also considered participants’ education level, current engagement in education and paid work, and ethnic background. A total of 79 were recruited and written informed consent was obtained from all participants.

### Data collection

**Fig 1:**
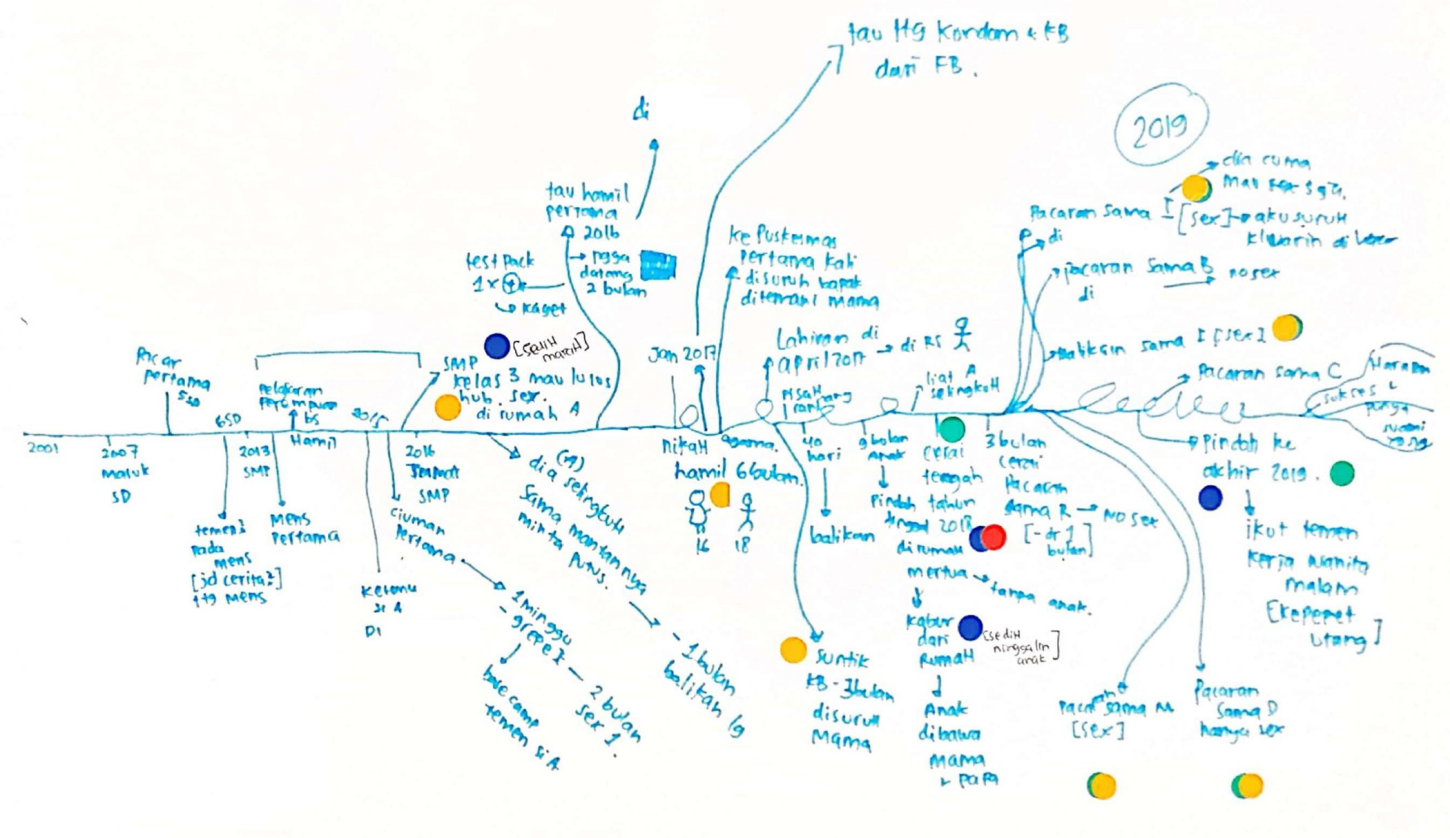
Sample timeline from Indonesia interviews.

Data collection was conducted in two phases. First, in-depth individual interviews were undertaken with young mothers in March 2021 to understand the drivers and pathways to adolescent pregnancy from their perspective. We used a participatory visual timeline approach, which has been used in prior research for mapping life histories [38,39] and understanding participants’ social contexts in relation to health and wellbeing outcomes [40–44]. Timeline interviews lasted 60 to 90 minutes. Researchers guided participants in generating a visual timeline on a sheet of paper, indicating key life events and milestones in their journey to becoming pregnant (Fig 1). This visual approach aimed to elicit participants’ interpretations of the questions, provide a creative and comfortable way for participants to tell their story, and build an image of participants’ perspectives and experiences in the context of their life story. Interviewers used a semi-structured topic guide to explore participants’ experiences of pregnancy, relationship with their partners, learning about sex and reproduction, sexual history, contraceptives, other relationships (e.g., family members, healthcare providers), and community and school influences. Key life events were added to the timeline throughout the discussion. Interviews were audio-recorded and transcribed verbatim into Bahasa Indonesia.

Second, following preliminary analysis of timeline interview data, in November to December 2021, we conducted follow-up phone interviews with 19 of the timeline interview participants who indicated their willingness to be recontacted for follow-up and consented to participate in follow-up interviews (11 girls from West Java, nine from Central Sulawesi). These interviews helped validate and clarify the study findings and interpretations and gather participants’ recommendations for programs and policy.

**Fig 2:**
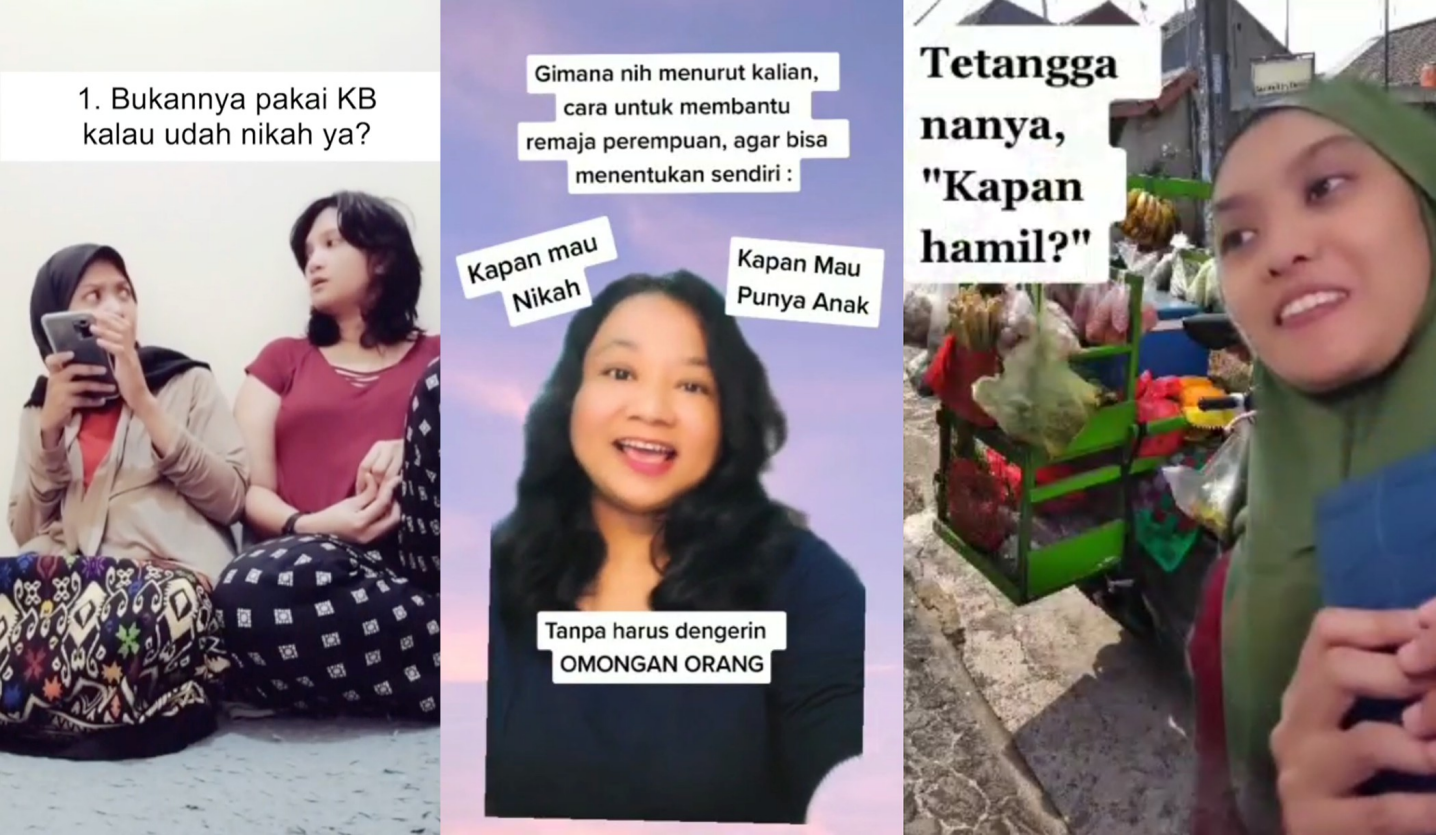
Stills of video clips developed to communicate preliminary study findings to follow-up interview participants.

To present the preliminary findings back to participants in an engaging way, the study team developed short video clips (Fig 2) on four major topics: knowledge about sex, reproduction, and contraception; contraceptive access and use; relationships, sex, and consent; and pregnancy and marriage. Participants were sent the video clips and given time to watch them before the follow-up interview. Researchers then used a semi-structured interview guide to validate and clarify findings and gather participants’ recommendations regarding strategies to support girls. Immediately after interview, interviewers completed a summary documenting participants’ feedback.

### Analysis

Analysis took an inductive approach, developing an understanding of drivers grounded in participant experiences. First, we transcribed audio recordings of interviews in the language that the interview was conducted in (Bahasa Indonesia), developed an initial framework, and summarised interviews in English based on this framework. Second, the researchers met to modify the framework and begin identifying themes/important drivers across participants. Third, multiple coders summarised the individual frameworks into a framework matrix in Microsoft Excel, adding themes as they became apparent. Finally, we developed candidate pathways and compared contributing drivers and characteristics across pathways through ‘cross-case’ analysis to develop a better understanding of the differences and similarities.

Findings from the follow-up interviews were integrated into final data analysis and helped to validate and clarify findings from the first phase of data collection. Likewise, participants’ recommendations gathered during follow-up were used to develop the study’s implications for practice.

### Ethical approvals

This study was granted research ethics clearance by the Alfred Health Ethics Committee (Project No. 14/21) and the Research Ethics Committee of the Atma Jaya Catholic University of Indonesia (No. 1248A/III/LPPM.PM.10.05/11/2020). The authors were not affiliated with the committees that provided ethical oversight.

Written informed consent was obtained from each participant before in-person interviews, while verbal consent was deemed sufficient for follow-up phone interviews. Adolescent girls invited to participate in the study were already parents or pregnant at the time of interview and had been asked to make their own decisions about medical care and interventions. Thus, study participants, including those aged below 18 years, were considered fully capable of understanding the involvement requested (interviews with potential follow-up interview), and as having the maturity to provide informed consent for their participation without the need for parental consent. This approach recognizes adolescents’ evolving capacities to make informed choices about their participation in research [45] and aligned with the recommendations of local research partners and country youth advisors. Participants were given either phone credit or e-money worth IDR100,000 (US$7) as a token of appreciation for their participation in the timeline interviews, and IDR 120,000 (US$8) to facilitate and as a token of appreciation for their participation in the follow-up phone interviews.

One participant was 15 years old at interview. This was reported to both ethics committees who determined that the data provided by the participant should be included in the study findings and recommended no further action.

### Inclusivity in global research

Additional information regarding the ethical, cultural, and scientific considerations specific to inclusivity in global research is included in the (S1 Checklist).

## Findings

### Profile of participants

Seventy-nine girls aged 15–21 years participated in the study. The median age was 18 years, with 38 participants from Central Sulawesi and 41 participants from West Java. Thirty-one participants were based in rural areas, 17 participants were based in peri-urban areas, and 31 participants were based in urban areas. Almost all participants were (or had been) married; fourteen were separated from or divorced their husbands, and one had been divorced but is now remarried. Five participants had never been married. Roughly half had completed at least middle school, about one-third had attended some senior high school, and 5% were high school graduates. One in five participants were engaged in paid work at interview. Almost all participants identified as Muslim, except three girls who identified as Christian.

### Six pathways to pregnancy

**Fig 3:**
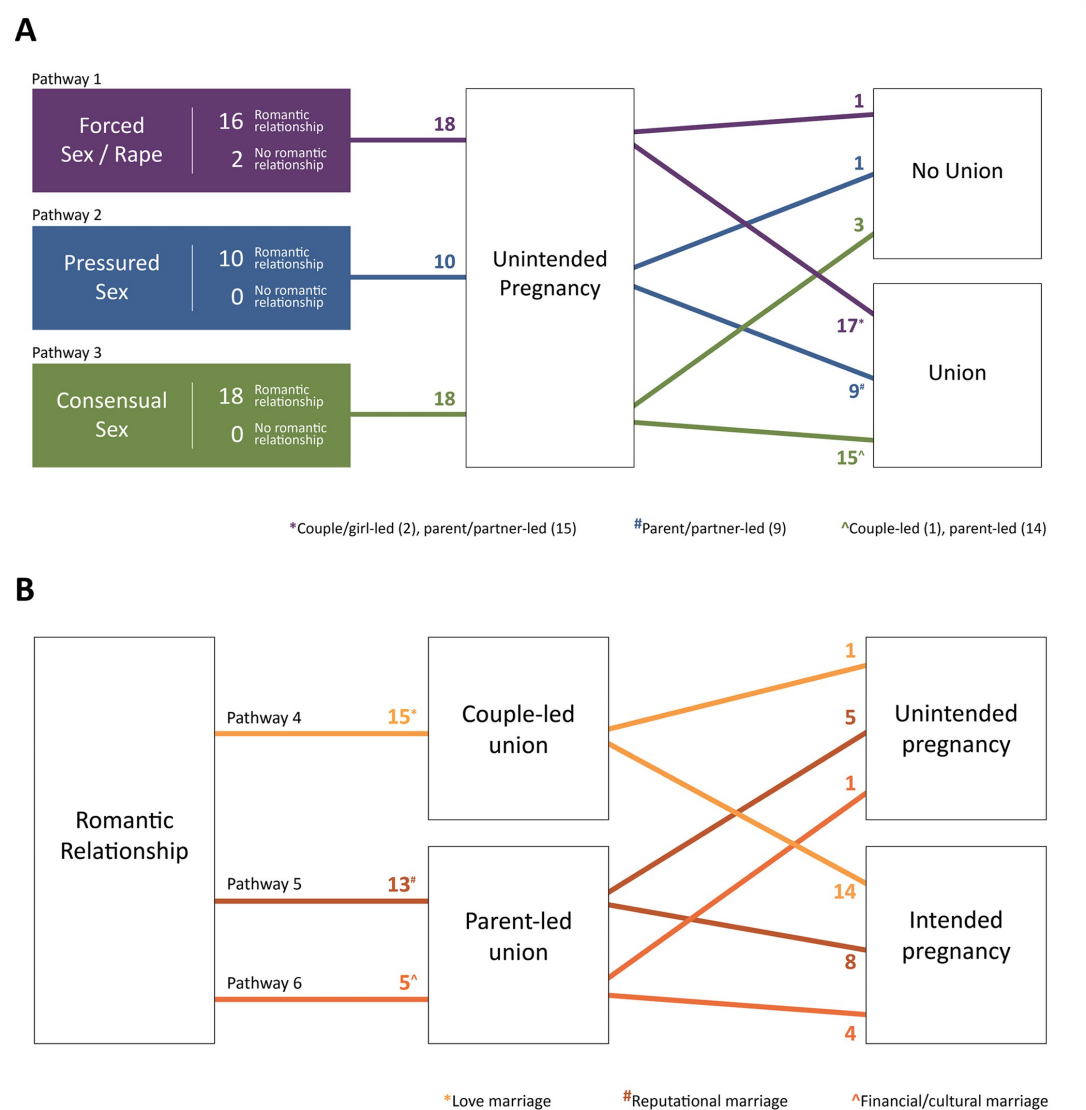
Pathways to adolescent (first) pregnancy in Indonesia.

We identified six distinct pathways to adolescent girls’ first experiences of pregnancy (Fig 3). The pathways were broadly differentiated by the timing of conception relative to marriage. Premarital pregnancy pathways diverged according to the nature of participants’ sexual relationship prior to pregnancy (these pathways include girls who had never been married), while post-marital pregnancy pathways were further differentiated based on the dominant motivation for marriage. We classified girls who did not marry the partner who got them pregnant under premarital pregnancy pathways as their pregnancies occurred outside a marital or cohabiting union and shared characteristics with those who moved forward with marriage. In analysing girls’ pathways, we also paid attention to girls’ sources of SRH information, any experience accessing and using contraception, and the outcome of the pregnancy (e.g., live birth, miscarriage, abortion). Each pathway is expanded upon below. Participants are referred to as ‘Participant XX’ to protect their privacy.

#### 1. Premarital pregnancy following experiences of forced sex

For 18 participants, the pathway to premarital pregnancy was characterised by experiences of forced sex (rape). Most participants belonging to this pathway were from urban and peri-urban areas in West Java; five were from Central Sulawesi (four rural, one urban). Many girls reported being drugged or drunk before experiencing forced sex; 11 girls indicated that they were raped while unconscious.

Nine participants became pregnant soon after being raped, the majority by their boyfriend; one girl became pregnant after being raped by someone she barely knew. Four of the nine girls reported being physically restrained or trapped in a room, and five were drugged or drunk at the time of rape.

The other nine participants experienced rape initially and had subsequent sex with their boyfriends which then led to pregnancy. Six of these girls reported being pressured/forced during subsequent sex–three described being made drunk before sex, two reported not wanting to have sex but being forced, while one was verbally pressured. As Participant 1 (age 18) from urban West Java recounted, *‘Every time I was brought to his boarding house*, *I was given a [spiked] drink*. *He promised not to do the same thing [after the first rape] but then it happened again*. *The third time*, *I didn’t want to go to his boarding house*, *he got mad and hit me*. *He forced me*.*’* Three girls reported having consensual sex with their boyfriends after being raped.

Only one participant reported that the person who raped her used a condom; seven reported that their partners used withdrawal. The rest reported no contraception and had no control over the decision. Some girls shared that their partner refused to wear a condom during subsequent sex due to discomfort or reduced pleasure.

Almost all girls eventually married the person who raped them (most through religious weddings, a few through religious and civil weddings). Marriages were most often initiated by the girls’ parents after learning of the pregnancy, although sometimes, the partners’ parents were also involved. In five cases, the boyfriend initiated religious marriage upon learning of the pregnancy. During follow-up, participants explained that girls who experience forced sex often felt that they could not leave that relationship because if it resulted in pregnancy, she would need the boyfriend to take responsibility and marry her. Girls also said rape victims often felt ‘broken’ afterwards and viewed themselves as undesirable to other men. Thus, some stayed with the partner who raped them, believing that he was the only person still willing to be in a relationship with them.

Notably in this pathway, even when pregnancy resulted from rape, it remained important to protect the girl’s reputation and avoid gossip through a swift marriage. Participant 2 (age 20) who lives in peri-urban West Java said, ‘*It just messed with your mind*. *The usual… neighbours talk*. *There are a lot of gossips*. *So*, *the sooner [the marriage]*, *the better*.*’* One participant reported initiating marriage with the help of her mother because she did not want to be pregnant without a husband. However, at the time of interview, only half the participants in this pathway were still married–the rest were divorced or separated. Few participants reported being happy with their marriage.

Half mentioned learning about puberty and reproduction at school (in science class) before pregnancy. After birth, 10 participants were using contraception, one participant was still pregnant at the time of interview, and one was planning to use contraception. The rest were not using contraception because they had divorced/separated from their husband and were not sexually active.

#### 2. Premarital pregnancy following unwanted and emotionally pressured sex

This pathway represents 10 participants who described not wanting to or being afraid to have sex but being emotionally pressured by their boyfriend. Most of these girls lived in rural or peri-urban areas and almost all lived in Central Sulawesi. Participants in this pathway were in a romantic relationship with someone who they met through friends or common social spaces or gatherings.

Participants’ first sex with their boyfriend occurred at varied lengths into the relationship, from within months to a year of dating. Girls described resisting their boyfriend’s sexual advances, but eventually relenting. Participants indicated that they did not want to have sex at the time but described different ways that their boyfriends persuaded them to have sex. These included giving gifts, getting angry or yelling, threatening to break-up, or promising to take responsibility if ‘anything happens’ (pregnancy).

Though the girls in this pathway had more agency in the decision to have sex compared to girls in the forced sex pathway, the advances and arguments made by their partners made it difficult for the girls to refuse sex. Participant 3 (age 18) who lives in rural Central Sulawesi did not really want to have sex with her boyfriend, but, ‘*I don’t dare to say no*. *I’m afraid that our relationship might not continue*.’ During follow-up, participants noted that girls expect boyfriends to request sex early in their relationship, but girls are likely to refuse initially to make sure that the boyfriend is committed to a relationship and not only with her for sex.

Most girls learned about sex from friends who shared their sexual experiences; only half learned about reproduction from classes at school, one of whom felt she was ‘too young to understand’ at the time. Many girls lacked awareness of contraceptive options, reporting that they had heard of condoms but did not know how to use them. Participant 4 (age 17) from rural Central Sulawesi recalled, ‘*I heard about using condoms*. *But I didn’t know its function*. *I just heard my friends talking about it*.*’* Participants reported that contraception decisions were made by their boyfriends. Six girls never used any form of contraception when having sex with their boyfriend and four used withdrawal.

All pregnancies in this pathway were unintended. About half of the girls considered or attempted abortion, and one was successful. Marriage was usually initiated by the girl’s parents shortly after the pregnancy became known. In a few cases, the boyfriend initiated the marriage with the support of the girl’s or his family. Like the other two premarital pregnancy pathways, marriage was expected by parents once the girl was pregnant. Participant 5 (age 17) from rural Central Sulawesi described that in her village, marriage needs to happen before the seventh month of pregnancy so as not to be ‘sinful’: ‘*People said after seven months… if you are not married yet*, *it could be seen as* haram *(sinful)*. *That’s why marriage needs to happen soon*.*’*

Three girls had religious marriages and two civil marriages. The type of marriage was unclear for the rest, and one did not marry because her relationship with her boyfriend ended after she had an abortion.

About half of those who were married during interview described their marriage positively and had started using hormonal contraceptives (e.g., pills, injections), some with support from family. One girl reported a difficult relationship and taking contraceptive injections secretly to avoid a second pregnancy.

#### 3. Premarital pregnancy following consensual sex

Eighteen girls’ pathway to pregnancy was characterised by consensual sex outside of union which then led to (usually) unintended pregnancy. Many resided in the peri-urban and urban areas of both study provinces, and their boyfriend was usually not more than three years older than them.

Compared to other pathways, more of these participants had prior romantic relationships. Many started dating the partner who got them pregnant when they were around 15 years old; typically meeting through friends or at school. The timing of first sex varied among couples, from within months to around a year of dating. First sex was usually not planned but wanted, and multiple participants described that they got ‘carried away’ (‘*terbawa suasana’*) when spending time together. Most couples practiced withdrawal or did not use contraception, though a few reported using condoms inconsistently. One girl experienced forced first sex perpetrated by an acquaintance but had consensual sex with a different person (her boyfriend) years later.

Most girls learned about sex when friends shared their sexual experiences, or they watched pornography together. Only half learned about reproduction in science or religious classes at school. Most participants were aware of some contraceptive options (e.g., condoms, pills) but did not know how to or had never attempted to use them. During follow-up, participants noted that it was ‘okay’ to use condoms but if a girl asks for contraceptive pills or injections ‘too early’ (i.e., while unmarried), people will become suspicious. A few reported that they did not know about contraception before they got pregnant. Some received more information about contraceptive options after birth or marriage. Participant 6 (age 18) from urban West Java recalled receiving extensive information on contraception through her youth group, but it was not clarified that contraception was also relevant before marriage so she dismissed the information as ‘not yet relevant’ for her: ‘*No*, *she [the presenter] did not explain that we could also use contraception before marriage*.*’*

Pregnancy was almost always unintended and around half of participants considered or attempted abortion. Some attempted abortion by drinking soda mixed with paracetamol or alcohol, or eating traditional herbs or foods believed to be abortifacient (e.g., pineapple). One participant reported a successful abortion, after ingesting a traditional herb. One participant reported that she and partner intended to get pregnant to facilitate marriage.

Most participants married the partner who got them pregnant shortly after learning of their pregnancy. Marriage in this pathway was usually initiated by the girl’s family although in some instances the boyfriend’s family was also involved. In two cases, the boyfriend initiated marriage along with the girl’s parents. One participant’s parents told her she did not have to get married and were supporting her to finish her education and raise her child alone. Two participants did not marry following an abortion and a miscarriage.

Marriage was often formalised through a religious ceremony due to the girl’s age. Many participants described not feeling ready for marriage but not having a choice. At the time of interview, most participants were still married and some described being satisfied with their marriage. About half were using contraception after their first or second birth.

#### 4. Couple-initiated marriage resulting in pregnancy

Fifteen girls entered a couple-initiated marriage. Typically, the boyfriend proposed, the girl accepted the proposal, and the couple discussed their engagement with their parents. Parents then supported (or at least accepted) the marriage, although in three cases, parents did not approve but the girl proceeded with the union. In three cases, the girl initiated the marriage. Parents were characterized as having much less control compared to other pathways and girls and their partners made their own decision to marry. These love marriages often occurred within one to two years in a romantic relationship, at a median age of 17 years. Couples were closer in age than in other pathways; most within five years. For half of participants, this was their first romantic relationship. Most girls got married to take the ‘next step’ in a romantic relationship–to express love or seriousness, or gain relationship security. One girl got married because she had ‘had enough of dating’ and wanted to have a child, while another was not keen on studying anymore; she saw that some of her friends were married and wanted the same for herself. Couples often met through friends or social media.

Most girls felt that they had finished enough schooling or did not want to continue. Many had stopped schooling prior to their marriage (some for financial reasons) or were happy to leave school following their boyfriend’s proposal.

Most girls in this pathway reported having sex for the first time after marriage. This sex was planned and desired by the couple and many more participants described being nervous but excited and enjoying sex than in other pathways. Only four participants in this pathway characterized sex as ‘a wife’s duty’.

Participants often had limited or no contraceptive knowledge before marriage; only three mentioned receiving any information about reproduction and contraceptives in science class or from teachers. Some received more information around the time of their marriage; others not until after their first birth (from midwives), although it is possible that participants may not have felt it was appropriate to admit familiarity with contraceptives. Most did not use any contraception before their first conception, and those who did, stopped using contraception because they wanted to get pregnant. As articulated in some timeline and follow-up interviews, there was an expectation of pregnancy soon after marriage and concerns about contraceptive use before the first birth making it difficult to conceive later on. For example, when Participant 7 was 16 and newly married, she was initially on injections as a contraceptive method but a midwife told her that she should ‘*not use it for too long’*.

In this pathway, most girls wanted to become pregnant and some were not using any contraception for this reason. All participants characterised their pregnancy as intended and none considered abortion. Participant 8 (age 17) from West Java explained, *‘When I got married the court told me to use contraception*, *but [*….*] I didn’t do it*. *I’m afraid my womb will dry if I did*.*’* She was very happy when she finally got pregnant four months after her wedding: *‘I was very happy when I finally got pregnant*, *because I was really waiting for it*. *I really wanted to have children*.*’* Most participants in this pathway decided to use contraception after their first birth.

#### 5. Reputational marriage resulting in pregnancy

For 13 participants, pregnancy occurred following a reputational marriage. Parents pushed for marriage when their daughter was in a romantic relationship and they were concerned that the girl might be gossiped about, vulnerable to premarital sex which would ruin her reputation, or at risk of pregnancy. As Participant 9 (age 17) from peri-urban West Java described, ‘*[I]t is better to marry young than to be pregnant before marriage*.*’*

In this pathway, participants’ boyfriends were similarly aged or up to nine years older. Reputational marriages typically occurred six months to a year after the girl started dating her boyfriend. For half of the participants, their relationship with their husband was their first romantic relationship; the rest had prior romantic relationships, but none had sex within these relationships. Like other post-marital pregnancy pathways, girls usually reported that their sexual debut occurred after marriage. Many described feeling apprehensive or shy, and half viewed sex with their husband as ‘a wife’s duty’.

Most girls who entered reputational marriages said they did not really want to get married, but their families usually insisted regardless of how the girl felt. Participant 10 (age 19) who lives in urban West Java said, *‘[My grandma said*,*] “Rather than dating*, *just get married because I [am] worried something might happen*.*” … But I don’t know how to feel*. *I feel confused*.*’* Half of the marriages were formalised through religious weddings as girls were not old enough to be married through a civil wedding–median age of marriage in this pathway was 16 years, whereas the minimum legal age of marriage in the country is 19 years [46–48]. About half of the participants stopped going to school because they got married, while others had already ceased schooling due to a lack of interest, financial constraints, or the desire or need to work.

About half of the girls described receiving some basic information about sex and reproduction from their teachers in school, usually through science class. A few girls also recalled learning about prohibited sexual acts (e.g., extramarital and premarital sex) in religious classes; one recalled being taught that a wife must obey her husband and not refuse sex when the husband asks. About half had limited awareness of contraceptive methods before marriage (usually from friends), while the others did not receive more information on contraceptives until around the time of marriage, pregnancy, or birth (usually from mothers and midwives). At the time of interview, only two participants reported using any contraception soon after marriage–one used an injection once on the advice of married friends but discontinued, while the other was supported by her mother to use injections until the couple desired a pregnancy.

All participants in this pathway got pregnant within a year of marriage. At follow-up, participants highlighted that some girls wanted to get pregnant soon after marriage because they felt bored or lonely, while others felt that having children was a way to feel ‘complete’ as a woman. They elaborated that it was considered ‘a woman’s duty’ to have a child (just as sex with their husbands was viewed as ‘a wife’s duty’). Participants in this pathway usually described a positive reaction to the pregnancy, with a smaller number of girls sharing that they reacted negatively. Two participants considered abortion; one of the two attempted by eating fruits that she had heard could cause abortion. However, both had been forbidden to pursue abortion by their husband who desired the pregnancy.

At the time of interview, almost all participants were still married; one participant was divorced following violence from her husband. Three participants reported concerns with partners’ drinking or behaviour towards them or their children, while some described satisfaction with their marriage and role as wife and mother. None were in school when they were interviewed. Four expressed their desire to finish school through the *Paket C* Program, three of whom reported that they were not allowed by their husbands. Most participants were either using contraception following their first birth (injection or intrauterine device), still pregnant, or planning to use contraception. Two participants reported concerns that husbands would not approve of contraceptive use and one reported fear of side effects.

#### 6. Financial marriage resulting in pregnancy

Five participants (aged 15–17 years) were married for a mix of financial and cultural reasons and became pregnant after marriage. This pathway was the least common and identified only among participants living in rural and peri-urban West Java. In three cases, parents negotiated marriage to provide for the girl, matching the participant with a prospective husband known to the family. The other two participants were in romantic relationships and agreed to marry their boyfriend primarily to reduce the financial burden on their families. Participant 11 (age 17) recounted, ‘*I said*, *“Okay*, *I’ll get married” because I want to stop burdening my parents*.’ Participants’ partners were significantly older (average age difference of 10 years) and earning income. Three participants noted that child marriage was common or accepted in their community.

All girls had their sexual debut after marriage and most reported feeling it was their ‘duty’ to have sex with their husband. Most participants had basic knowledge about puberty and reproduction from science and religious classes at school. Yet, before marriage, they reported having limited or no knowledge about contraceptives. Reasons for not using contraceptives varied, including fear of side effects (e.g., the belief that hormonal contraceptives could ‘dry the womb’) and partner’s preference for withdrawal. At the time of interview, most girls had some knowledge about hormonal and barrier contraceptive methods, learned mostly from friends and the Internet (e.g., Facebook).

Most participants in this pathway described their pregnancy as intended, although in three cases, it was largely the husband who wanted to have children. Participant 12 (age 20) recalled her conversation with her husband: *‘I already told him [I didn’t want a baby yet]*, *but he told me*, *“What about the others around us*? *Over there*, *already [with child]*. *My younger schoolmate there*, *also already [with child]*.*”‘*

Two participants’ initial financial marriages ended in divorce; one remarried and was pregnant to her second husband. Others reported mixed satisfaction with their current relationship. Three participants were currently using or previously used contraception to delay a second pregnancy.

## Discussion

Our study aimed to understand the pathways to adolescent pregnancy in Indonesia and identify contributing drivers. Through analysis of our timeline interviews, we identified six pathways which were differentiated by timing of pregnancy relative to union, main motivation for union, and nature of the sexual relationship preceding pregnancy. Three pathways captured the experiences of girls who conceived after marriage motivated by her financial circumstances, parents’ fears for her reputation, or initiated by the couple wanting to move to the next step in their life together. A further three pathways captured the experiences of pregnancy prior to any marriage following consensual, pressured, or forced sex. Our identified pathways highlighted drivers of adolescent pregnancy which are common across the groups, including: the acceptability of child marriage, harmful gender norms that stigmatise adolescent sexuality and premarital sex and support gender-based violence, and late comprehensive contraceptive information and support.

### Acceptability of child marriage and stigma of premarital pregnancy

Community acceptance of child marriage facilitated the three post-marital pregnancy pathways. Child marriage was viewed as a justifiable means to gain greater financial security for a girl and reduce economic pressure on her family. Although this was a small proportion of our sample, this finding is consistent with other research findings. Strong social norms in support of child marriage remain prevalent in some Indonesian communities [49,50]. A study in South Sulawesi found that one-fourth of adults and one-third of adolescents agreed with the view that girls aged 18 and above who are not yet married are a burden to their families [51]. Acceptance of child marriage has been found more likely among women with lower education and based in rural areas [52].

Child marriage is also viewed as a way to regulate ‘immoral’ behaviour and protect the honour of a girl and her family [49,53]. In line with other studies [31,49,54], we found that some parents pre-emptively married off their daughter to avoid the social consequences of an unmarried girl getting ‘too close’ to a boy/man and potentially engaging in ‘forbidden’ (premarital) sex. Meanwhile, for young couples in romantic relationships, love marriage was viewed as the most acceptable way of taking the next step in their relationship while adhering to religious and sociocultural ideals.

In the event of a premarital pregnancy, participants and their families viewed marriage as the only socially acceptable ‘solution’. Nationally, about one in four Indonesian women aged 20–24 conceive before marriage [20], and the dominant view that marriage is the only way to ‘legitimise’ a premarital pregnancy in Indonesia has been well documented [55–57]. In our study, when negotiating sex, boyfriends often suggested they would take responsibility for premarital pregnancy. Participants also highlighted that a girl’s reputation is compromised if the man who got her pregnant refuses to take responsibility through marriage. Our findings are consistent with other studies [36,58], that once pregnant, girls often had little or no control over the decision to marry [36,58].

Despite this common view of marriage as a ‘solution’, less than a third of participants in the premarital pregnancy pathways who were married described their marriage positively at interview. Separation and divorce were common among those in the forced sex pathway. While divorce is allowed in Indonesia, it is viewed negatively; in particular, divorced girls/women face considerable stigma in society [59,60]. Our findings suggest that child marriage following premarital sex or pregnancy may not yield the positive relationship or life outcomes that parents and girls hope for.

### Partner and family expectations of pregnancy following marriage

Partner and family expectations of pregnancy following marriage discouraged contraceptive use and contributed to many pregnancies. Some participants expressed that girls wanted to get pregnant after marriage because it was expected of them, could prevent boredom or loneliness, and was a way to become ‘complete as a woman’. These findings affirm what Parker referred to as the ‘sacred triangle of sex, marriage, and reproduction’ [35]. In Indonesia, aside from regulating sexuality, the main goal of marriage is reproduction, and young people are brought up to aspire to these ideals [35]. Our findings also resonate with those of Bennett and Arai, that for girls from resource-constrained backgrounds, motherhood can present a ‘meaningful life option’ [61], especially when being a mother is a highly valued source of social identity [54].

Although marriage was viewed as the acceptable time for couples to engage in sex and for girls to receive information about sex and contraception, married girls who did not want to get pregnant were not supported to use contraceptives. Some were given contraceptive information by mothers, healthcare workers, or marriage officiants, but it was rarely used, often because the husband or the couple’s families desired a pregnancy. This helps explain the quantitative findings in other research that most married adolescents do not use contraceptives, and those who do, typically do so after the first child for birth spacing and limiting [62]. Consistent with this, acceptability of contraception after the first birth was clear across our sample. Many participants received information and support from healthcare providers on modern methods after their first birth.

### Harmful gender norms and sexual violence

Many participants’ narratives featured harmful norms that were supportive of traditional gender roles around sex, including male dominance and female submission. Girls in financial and reputational marriage pathways often reported having sex with their husbands because they considered it ‘a wife’s duty.’ This was also observed in a study in Lombok, Indonesia which noted the prevalence of idealized sexual scripts that depicted a husband’s right to have sex with his wife and a wife’s obligation to fulfill her husband’s sexual desires [31]. While male sexuality is expected, female sexuality is highly stigmatized, and sex within marriage is presumed to be consensual and intended mainly for reproduction [31,35]. Bennett further argued that these traditional constructions of femininity and female sexuality perpetuate early marriage practices which are in turn, closely associated with adolescent childbearing [54].

Harmful gender norms supportive of gender-based violence were also evident in the narratives of girls who got pregnant following unwanted/pressured or forced sex. It is important to note that despite strong social disapproval of unmarried girls engaging in intimate relations [30,49], most girls in our study who followed pathways to pregnancy outside of union had been in genuine romantic (dating) relationships from a few months to over a year when sex occurred. In girls’ narratives of unwanted/pressured sex, girls resisted their boyfriends’ repeated advances but eventually relented, many because they cared for their boyfriend and were afraid that he would become upset or end the relationship if they refused; this was indicative of inequitable gender relations within dating relationships. Meanwhile, our participants’ depictions of male partners’ persistence in convincing or pressuring girls to have sex, and in more extreme cases, raping girls who were drunk or drugged, were illustrative of male partners’ sense of sexual entitlement. This can again be linked to findings from the 2017 Indonesia DHS, that 16% of never-married female adolescents reported having sex for the first time because they were ‘forced by (their) partner’ [18]. Other studies in Indonesia have noted similar power dynamics between adolescent girls and their boyfriends, with girls unable to refuse their partners’ requests for sex for comparable reasons [30,35], and girls’ apparent lack of skills to negotiate [36,54]. Toward preventing gender-based violence, there is a need for community interventions that promote gender equality by transforming gender norms that perpetuate the subordination of women and girls and repress female sexuality [63,64]. In addition, as others have emphasized, there is an urgent need to establish policy mechanisms that protect girls from sexual violence and enable victims of sexual violence to pursue legal action [36]. It will be critical to ensure that the newly passed anti-sexual violence law [65]–which outlaws sexual violence both within and outside marriage, and requires abusers to pay restitution and authorities to provide mental health and psychosocial support for victims–is enforced and bolstered by clear implementation protocols on the ground.

In a divergent set of cases, adolescent girls engaging in consensual premarital sex and couple-initiated marriages described more equitable sexual relationships and greater satisfaction with their eventual marriage. However, these couples were still impacted by stigma surrounding premarital sex, and the expectations that the boyfriend would take responsibility for contraception or subsequent pregnancy.

### Sexual and reproductive health information and access to services

Many participants belonging to the premarital pregnancy pathways expressed that they did not want to be married or pregnant yet. Girls did not expect to get pregnant, even those who knew that sex could lead to pregnancy, indicating the need for improved access to SRH information and contraception. Most participants mentioned receiving some (not always accurate) information about sex from their friends and the Internet, and limited, often only basic information about puberty and reproduction in classes at school. While many had heard of condoms, they did not fully understand what they were or how to use them [see also 30], and viewed condom procurement and use as the responsibility of their boyfriend. This can be partly explained by findings from the 2017 Indonesia DHS, that most youth discussed reproductive health with their friends and rarely learned about family planning in schools [18]. DHS data also show that knowledge of the fertile period was poor among women across age groups, but especially so among girls aged 15–19 years [19], indicating a need for improved access to SRH information. Currently, there is no law or policy regarding the provision of sexuality education for young people in Indonesia [66], and recent reviews have emphasised that no country in the Asia-Pacific region currently delivers comprehensive sexuality education (CSE) in line with international standards [16].

Furthermore, our participants reproduced attitudes typically held by parents and teachers that when it came to unmarried young people, information about sex and contraception was ‘not yet needed’ or appropriate. This was particularly important for the group experiencing consensual premarital sex, where improved access to contraceptives may have prevented their pregnancy. During follow-up, participants highlighted that the sex education they received did not clarify that contraception can also be used before marriage. Thus, participants often dismissed contraception information as irrelevant.

Evidence shows that sexuality education is best started early (beginning at age 5 years) and most effective at reducing adverse SRH outcomes when it incorporates a focus on gender equality and human rights [67]. Our findings underscore the need to strengthen Indonesia’s sexuality education curriculum at both primary and secondary school levels by making it mandatory beginning in primary school, and ensuring that the curriculum covers all core concepts specified in the UNESCO International Technical Guidance on Sexuality Education [67]. In response to our findings on sexual violence, it will be particularly important for the sexuality education curriculum to prioritise topics related to gender, respectful relationships, decision-making, and consent. This will require clear policies and laws regarding the provision of age- and developmentally-appropriate CSE to children and adolescents, complemented by access to adolescent-responsive SRH services (regardless of marital status), consistent with the Indonesian government’s commitments to the 2030 Agenda for Sustainable Development, International Conference on Population and Development Programme of Action, and Family Planning 2030 to fulfil sexual and reproductive health and rights for all [68–70].

Across pathways, many girls learned more about their modern contraceptive options from midwives after their first birth, along with support to initiate use. However, the overall lack of accurate information and prevalence of myths about contraception combined with power imbalances within relationships all affected participants’ motivation to access and use contraceptives and their confidence in negotiating contraceptive use with their partners. Contraception decisions were more often made by boyfriends or husbands and parents/in-laws after marriage, consistent with past studies [71]. A few couples used condoms initially but discontinued when the boyfriend or husband no longer wanted to use condoms.

Many participants who had a premarital conception considered or attempted abortion, usually through ineffective methods learned through word of mouth. Only three participants successfully aborted. Although abortion is illegal and highly stigmatized, knowledge of abortion was common among our sample, and this is consistent with a quantitative study that found that many Indonesian women seek induced and often unsafe abortion [53].

### Strengths and limitations

Conducting the study in two provinces and across rural and urban settings in Indonesia allowed us to capture a range of experiences and develop a better understanding of Indonesian girls’ lived realities of adolescent pregnancy. However, further qualitative inquiry in other geographical areas would be valuable, as Indonesia is widely diverse.

The individual pathways to adolescent pregnancy were outlined during timeline interviews with participants where each determined for themselves the important milestones in their personal story. The follow-up interviews after preliminary analysis served as a member-check to validate the findings, check participants’ understanding, and also ‘fill in the gaps.’

The phone-based approach to the follow-up interviews was used in lieu of in-person group workshops in response to COVID-19 exposure risk and restrictions that were in place during the conduct of the study. An in-person follow-up may have yielded different insights to complement the main data collection.

This study only engaged with girls, and many of our findings relate to the expectations of their partners. Further studies are required to help shed light on the perspectives of young men, parents, and service providers, such as midwives and teachers.

We recommend future quantitative research that explores the prevalence of pregnancy because of rape and the prevalence of relationship outcomes (e.g., intimate partner violence, separation/divorce) across the different pathways to adolescent pregnancy. Future qualitative research could also focus on developing a better understanding of male partners/husbands’ motivations for pregnancy (i.e., why it is important to them to have children soon after marriage), their disapproval/dislike of contraceptive methods, and whether spousal age gap influences couples’ contraceptive use.

### Conclusion/Implications for practice

Our findings suggest that reducing unintended adolescent pregnancy is a critical step toward eliminating child marriage. This includes attending to the harmful gender norms, increasing girls’ communication skills and agentic power in romantic relationships, engaging men and boys in critically examining gendered sexual scripts, and establishing more equal power dynamics in relationships. It will likewise be important for interventions to highlight ‘a woman’s worth’ beyond childbearing. Toward this end, it will be important to engage girls’ families and community members, increase access to and support for alternative trajectories to girls’ self-fulfilment, and focus on developing gender transformative norms regarding how men view and treat women in relationships. Finally, it is imperative to establish and enforce clear legal and social consequences for perpetrators of gender-based violence.

We identified the need to ensure that sexuality education provided to adolescents is comprehensive and gender transformative and incorporate in-depth discussions and life skills training. We also observed a need to clarify–at all levels of social life (i.e., relationships, community, society)–messaging around the use of contraception, including emphasising the relevance of premarital usage of contraception and addressing the myth of infertility side effects. Increasing accessibility and availability of contraception for young and unmarried women by addressing the legal, structural, and community-level barriers to access has the potential to increase premarital contraceptive use.

Our findings highlight many drivers of adolescent pregnancy in Indonesia, and the diversity of girls’ lived realities. Participant pathways represented six different typologies, all of which may be best addressed by different intervention approaches. Future research should identify effective interventions to combat our identified drivers of adolescent pregnancy, and provide quantitative data on the prevalence and contribution of the identified pathways to enhance policy and programme planning.

## Supporting information

S1 Checklist (DOCX)
